# Comfort and Data Sharing With Artificial Companion Robots Among an Online Cohort

**DOI:** 10.1093/geroni/igab046.1025

**Published:** 2021-12-17

**Authors:** Clara Berridge, Yuanjin Zhou, Julie Robillard, Nora Mattek, Sarah Gothard, Jeffrey Kaye

**Affiliations:** 1 University of Washington, Seattle, Washington, United States; 2 University of British Columbia, Vancouver, British Columbia, Canada; 3 Oregon Health & Science University, Portland, Oregon, United States

## Abstract

Results from a June 2020 survey on comfort with two forms of artificial companion (AC) robots in normal compared with pandemic times will be presented. 1,082 adults age 21-92 (mean 64) completed the online survey for a response rate of 45%. Significantly greater comfort is reported with small AC robots relative to larger human-shaped robots in both normal and pandemic times. In bivariate and adjusted models, younger age and male gender were most commonly associated with greater comfort with AC robots. Most participants (68.7%) did not think an AC robot would make them feel less lonely. About half (52.8 %) of the participants reported that they probably or definitely would want their facial expressions to be read, while a minority (15.0%) were at least somewhat comfortable with AC robots recording their conversations. The most common person participants wanted these data types shared with is themselves, a spouse/partner, and medical provider.

